# Editorial: Insights in cardiovascular and smooth muscle pharmacology: 2023

**DOI:** 10.3389/fphar.2024.1544594

**Published:** 2025-01-06

**Authors:** Sönke Schildt, Daniel Reichart, Simon Lebek

**Affiliations:** ^1^ Department of Internal Medicine II, University Hospital Regensburg, Regensburg, Germany; ^2^ Department of Medicine I, Ludwig-Maximilians-Universität Munich, Munich, Germany; ^3^ Gene Center Munich, LMU Munich, Munich, Germany; ^4^ DZHK (German Centre for Cardiovascular Research), Partner Site Munich Heart Alliance, Munich, Germany

**Keywords:** cardiovascular disease, heart failure, arrhythmias, translational medicine, pharmacology

Cardiovascular diseases remain the most common cause of death worldwide (Roth et al., 2020). Although new therapies have been developed in recent decades, there is still an urgent need for new approaches and innovative research. In this Research Topic of Frontiers in Pharmacology, we immerse in relevant aspects and present new research findings, guiding you through potential approaches and novel aspects in cardiovascular medicine.

Worldwide, 26 million patients suffer from heart failure, approximately half are diagnosed with heart failure with preserved ejection fraction (HFpEF) (Savarese and Lund, 2017; Lebek et al., 2021). Although several drugs with prognostic relevance are available for heart failure with reduced ejection fraction (HFrEF), such treatments are still lacking for HFpEF, underlining the urgent need for new therapeutics (Krittanawong et al., 2024). Highlighting the pathway’s molecular mechanisms, the review of Jiang et al. outlines the various factors triggering fibroblast activation, and leading to excessive remodeling and subsequent HFpEF. By focusing on JAK/STAT3, the authors present potential therapeutic approaches to counteract fibrosis, providing insights for future research on anti-fibrotic treatments. This review gives a valuable overview of the complexity of cardiac fibrosis and presents ideas for new, targeted therapies to combat this challenging condition.

Also closely connected and in clinical practice often missed comorbidity in HFpEF are sleep disorders (Wester et al., 2023). Arrhythmias in this context are addressed by Hegner et al., investigating the connection between sleep apnea syndrome and atrial arrhythmias. The study vividly shows that the increased production of reactive oxygen species due to obstructive sleep apnea leads to cellular sodium overload and induction of cellular arrhythmias. These novel insights into the mechanisms of arrhythmias in obstructive sleep apnea provide evidence for the necessity of potential approaches to targeted therapy in this area.

Twenty years after the discovery of PCSK9 and its effects on LDL cholesterol metabolism, its inhibition by monoclonal antibodies has become one of the most effective methods for lowering LDL levels and hereby reducing the progress of cardiovascular diseases (Abifadel et al., 2003; Cohen et al., 2005; Zendjebil and Steg, 2024). Beyond its central role in liver LDL receptor metabolism, PCSK9 is also present in cardiac, cerebral, renal, and other tissues, where it supports essential physiological functions. The review from Lu et al. examines the protective role of PCSK9 in extrahepatic tissues, highlighting risks of deficiency, such as lipid buildup, mitochondrial dysfunction, and insulin resistance. By analyzing experimental and clinical findings, it provides insights into the complex effects of PCSK9 inhibition, encouraging a balanced view on its therapeutic potential.

The renal function reflects another major player in the physiology and pathophysiology of the cardiovascular system. Here, the study by Toth et al. vividly highlights how the inhibition of hypoxia-inducible factor 1α (HIF1α) by Daprodustat is linked to vascular calcification. Atherosclerosis is a significant complication, particularly in patients with end-stage renal disease and on dialysis, making this study an important step forward in the understanding of the underlying pathomechanisms (Marando et al., 2024). In addition, Yu et al. provides detailed insights into how Endothelin-1 receptor (ET-1) antagonists could be used to regulate blood pressure and fluid balance, which is particularly important for the treatment of cardiovascular and kidney diseases. The developed model could help to modulate the targets and effects of ET-1 more precisely and minimize side effects associated with ETA antagonists such as fluid retention.

And if nothing else helps? The article by Von Bibra and Hinkel provides an intriguing overview of current research on stem cell-based remuscularization transplantation. The focus is on translational application and study execution in non-human primates. It offers a practical description of the advantages and disadvantages of various approaches, providing not only a solid overview of the current state of research but also suggesting possibilities for clinical translation. Although the path to a lab-grown heart is still distant, initial steps leading to independence from transplants are already in clinical testing.

But where might future cardiovascular medicine develop in the coming years? Even though cardiovascular research brought several new and powerful drugs into clinical practice (e.g., gliflozins or mavacamten), patients’ prognosis is still limited and comparable to that of cancer patients (Ponikowski et al., 2014; Roth et al., 2020). This is because current treatments are either ineffective in certain patient populations (HFpEF vs. HFrEF) or associated with severe adverse side effects (Heidenreich et al., 2022). The latter might be either due to unspecific off-target binding of the compound or due to on-target binding in another tissue where the target protein is not necessarily pathogenic (Pellicena and Schulman, 2014; Nassal et al., 2020). Another major challenge in cardiovascular medicine is the poor compliance of patients to take their prescribed medication, which further decreases with every extra pill they need to take (Kulkarni et al., 2006; Gupta et al., 2017). This highlights the urgent need for precise and tissue-specific approaches that ideally confer sustained therapeutic benefits. We previously demonstrated that this can be achieved by CRISPR-Cas gene editing (Lebek et al., 2023a; Lebek et al., 2023b; Lauerer et al., 2024; Reichart et al., 2023).

In conclusion, this Research Topic underlines the urgent need for research in the field of cardiovascular medicine, which will provide new targets and potential therapeutic strategies. Future therapies will focus on minimizing side effects while enhancing efficacy for long-lasting therapeutic benefits.
